# One-step synthesis of 1,6-hexanediamine modified magnetic chitosan microspheres for fast and efficient removal of toxic hexavalent chromium

**DOI:** 10.1038/s41598-018-29499-z

**Published:** 2018-07-23

**Authors:** Rui Yue, Qiumeng Chen, Siqi Li, Xiaodan Zhang, Yuming Huang, Ping Feng

**Affiliations:** Key Laboratory of Eco-environments in Three Gorges Reservoir Region (Ministry of Education), College of Chemistry and Chemical Engineering, Southwest University, Chongqing, 400715 China

## Abstract

Here, we reported a novel one-step hydrothermal route for the facile synthesis of 1,6-hexanediamine functionalized magnetic chitosan microspheres (AF-MCTS), which were characterized by TEM, FT-IR and XPS to look into its morphology, surface functional groups, and adsorption mechanism of Cr(VI) from the aqueous solution. Cr(VI) adsorption on AF-MCTS as a function of contact time, Cr(VI) concentration, pH, and ionic strength was investigated. The adsorption process follows the Langmuir isotherm model and pseudo-second-order kinetic model. The AF-MCTS exhibited high performance for Cr(VI) removal with very fast adsorption rate (reaching equilibrium within 5 min) and high adsorption capacity (208.33 mg/g), which was 1.1 to 12 times that of other chitosan-based adsorbents. Cr(VI) adsorption onto AF-MCTS was an endothermic and spontaneous process. The recovery and reuse of AF-MCTS was demonstrated 11 times without obvious decrease in adsorption capacity. Mechanism study suggested that –OH rather than –NH_2_ groups in AF-MCTS were the electron donors for reducing Cr(VI) to Cr^3+^. Consumption or addition of H^+^ could trigger the reversible supramolecular coordination between Cr^3+^ and chitosan. Given the easy preparation, low cost, and remarkable performance, AF-MCTS composite is expected to show promising potential for the practical application in removing toxic Cr(VI) from aqueous media.

## Introduction

Chromium mainly comes from a variety of industry activities^1^. These include leather tanning, plating and water cooling^1^. Chromium exits as two common oxidation states in aqueous media, namely Cr^3+^ and Cr(VI). In contrast to Cr^3+^, Cr(VI) is highly toxic, soluble, and mobile. Importantly, Cr(VI) likely causes carcinogenicity and mutagenicity^2^ for living being because it possesses a strong oxidizing characteristic. Thus, its elimination from liquid waste before its discharging into environment is highly demanded. Various technologies were exploited for Cr(VI) elimination from aqueous media. These include adsorption^3^, ion exchange^4^, chemical reduction^5^, adsorption-reduction^6^, electrocoagulation^7^, constructed wetland-based technologies^8^, and so on. Among various approaches, adsorption is believed to be one of promising approaches due to its high performance^9^.

Chitosan (CTS) is a kind of vital natural polymers, which was obtained from chitin via the deacetylation process^10^. It is low cost and abundant in nature. Also, CTS is a natural eco-friendly material^11^ that possesses the adsorption ability for heavy metal ions through complexing with the hydroxy and amino groups^10,12^. On the other hand, these functional groups in CTS also have good affinity to anion ions such as HCr_2_O_7_^−^ and Cr_2_O_7_^2−^. Therefore, these characteristics make CTS a favorable bioadsorbent, which could be used for removing heavy metals in aqueous systems^10^. However, unmodified CTS exhibits poor adsorption capacity to Cr(VI)^13^ due to the limited adsorption sites caused by 3D ordered crystal architecture possessed by native CTS^14^.

Recently, to enhance its adsorbing ability for Cr(VI), various methods for the modification of CTS have been demonstrated. These include titanium cross-linked chitosan composite^15^, zirconium cross-linked chitosan^16^, chitosan-Fe(III) complex^17^, ethylenediamine modified chitosan microsphere^18,19^, *n*-butylacrylate grafted chitosan^20^, and so on. What’s more, the magnetic nanoparticles (MNPs) have continued to draw considerable interests for their large surface areas and simple magnetic separation via a magnet. Therefore, the hybrid adsorbents combined with MNPs and CTS have received growing interest for Cr(VI) removal^21^. For instance, through microemulsion process, Chen *et al*. synthesized chitosan/montmorillonite–Fe_3_O_4_ hybrid for Cr(VI) adsorption^22^. Under optimum pH of 2.0, the chitosan/montmorillonite–Fe_3_O_4_ could adsorb 58.8 mg/g Cr(VI) (the theoretical adsorption amount). Li *et al*. reported the synthesis of the magnetic *β*-cyclodextrin–CTS modified graphene oxide (GO) for removing Cr(VI) with a maximal adsorption amount of 67.66 mg/g^23^. Xiao *et al*. reported an eco-friendly method to synthesize the chitosan-coated MnFe_2_O_4_ nanoparticles (NPs)^24^. However, only 35.32 mg/g maximal adsorption amount was acquired for Cr(VI). A simple method to synthesize magnetic CTS NPs by co-precipitation through epichlorohydrin cross-linking process was proposed, and the resultant magnetic CTS showed a 69.4 and 55.80 mg/g of the maximum adsorption capacity for hexavalent chromium^25,26^. In a recent work, Liu’s group used the emulsion crosslinking technique to prepare the polyethylenimine modified magnetic SiO_2_–CTS microspheres through four-step procedure, showing enhanced Cr(VI) uptake amount of 236.4 mg/g^27^. Unfortunately, the adsorption process was relatively slow (60–360 min)^24,26,27^. In order to improve the adsorption rate, Hu *et al*. prepared the ethylenediamine-functionalized glutaraldehyde-crosslinked magnetic CTS adsorbent for Cr(VI) to achieve adsorption equilibrium within 6–10 min^18^. While the adsorption amount was relatively low (51.81 mg/g). This was probably caused by the depletion of amino groups in glutaraldehyde crosslinking process. In 2014, Debnath *et al*. succeeded in preparation of the magnetic CTS–GO nanocomposite to remove Cr(VI) with a adsorption amount of 101.6 mg/g at optimum pH of 3.0^28^. Also, we used the CTS−Fe(III) hydrogel to absorb Cr(VI) with maximal adsorption amount of about 170 mg/g in our previous work^29^. However, tedious procedures were used for the preparation of such CTS−Fe(III) hydrogel^29^.

Above examples indicated that the preparation of these magnetic chitosan adsorbents was relatively complicated, usually involving several steps, including synthesis and surface functionalization of magnetic NPs, and coating of magnetic NPs by chitosan (Table S1, Supporting Information). Furthermore, in most cases, chemical cross-linking was adopted to enhance the acid resistance and mechanical property of CTS (Table S1, Supporting Information), which suffered from some drawbacks, such as consumption of -NH_2_ groups in CTS during the cross-linking reaction, leading to decrease of Cr(VI) adsorption (Table S1, Supporting Information). Hence, it will be interesting to develop a facile and simple one-pot synthesis of magnetic chitosan for quickly and efficiently removing Cr(VI) from aqueous solution. Here, we reported a novel one-step hydrothermal route for the facile synthesis of amino-functionalized magnetic chitosan microspheres (AF-MCTS), yielding a composite adsorbent with very fast adsorption rate (5 min) and high uptake amount (above 200 mg/g) for Cr(VI) removal. The resultant AF-MCTS was characterized by TEM, FT-IR, VSM as well as XPS. The Cr(VI) removal mechanism was discussed.

## Results and Discussion

### Characterization of the adsorbent materials

**Figure 1:**
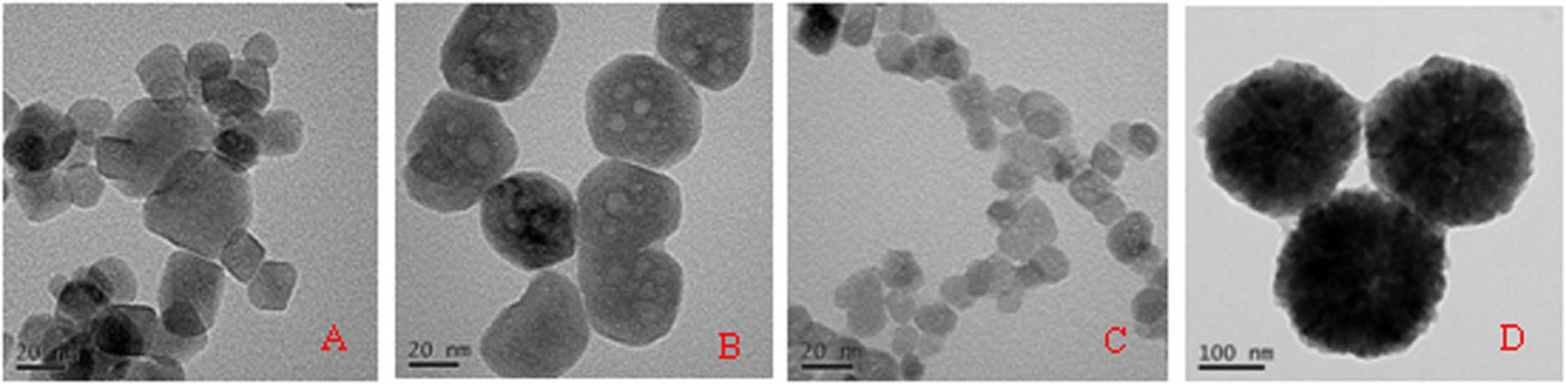
TEM images of AF-Fe_3_O_4_ (**A**), AF-MCTS-0.5 (**B**), AF-MCTS-4 (**C**), and MCTS particles (**D**).

The morphologies of the materials are characterized by TEM. Figure 1 presents TEM photos of the AF-Fe_3_O_4_, AF-MCTS-0.5, AF-MCTS-4, and MCTS, respectively. The AF-Fe_3_O_4_ particles are irregular in shape and have a size of 20~40 nm (Fig. 1A). After the CTS functionalization, the morphology of the obtained AF-MCTS was still irregular; furthermore, the obtained AF-MCTS particles were more uniform. However, the average size of the obtained AF-MCTS decreased from about 50 nm (Fig. 1B) to about 20 nm (Fig. 1C) as CTS amount varied from 0.5 to 4.0 g. It is noted that the size of MCTS (about 250 nm, Fig. 1D) was much bigger than that of AF-MCTS, indicating key roles of both CTS and 1,6-hexanediamine in controlling the size of the resultant particles. Also, the EDS provides the superficial chemical compositions of the AF-MCTS. The EDS spectra and atomic ratio of corresponding elements in AF-MCTS composite are shown in Fig. S1 in Supporting Information. As can be seen, C, N, O, and Fe elements were detected in the AF-MCTS composite. The atomic concentration of N in AF-MCTS is 7.99%, which is higher than that of CTS with 7.54% nitrogen content^30^.

**Figure 2:**
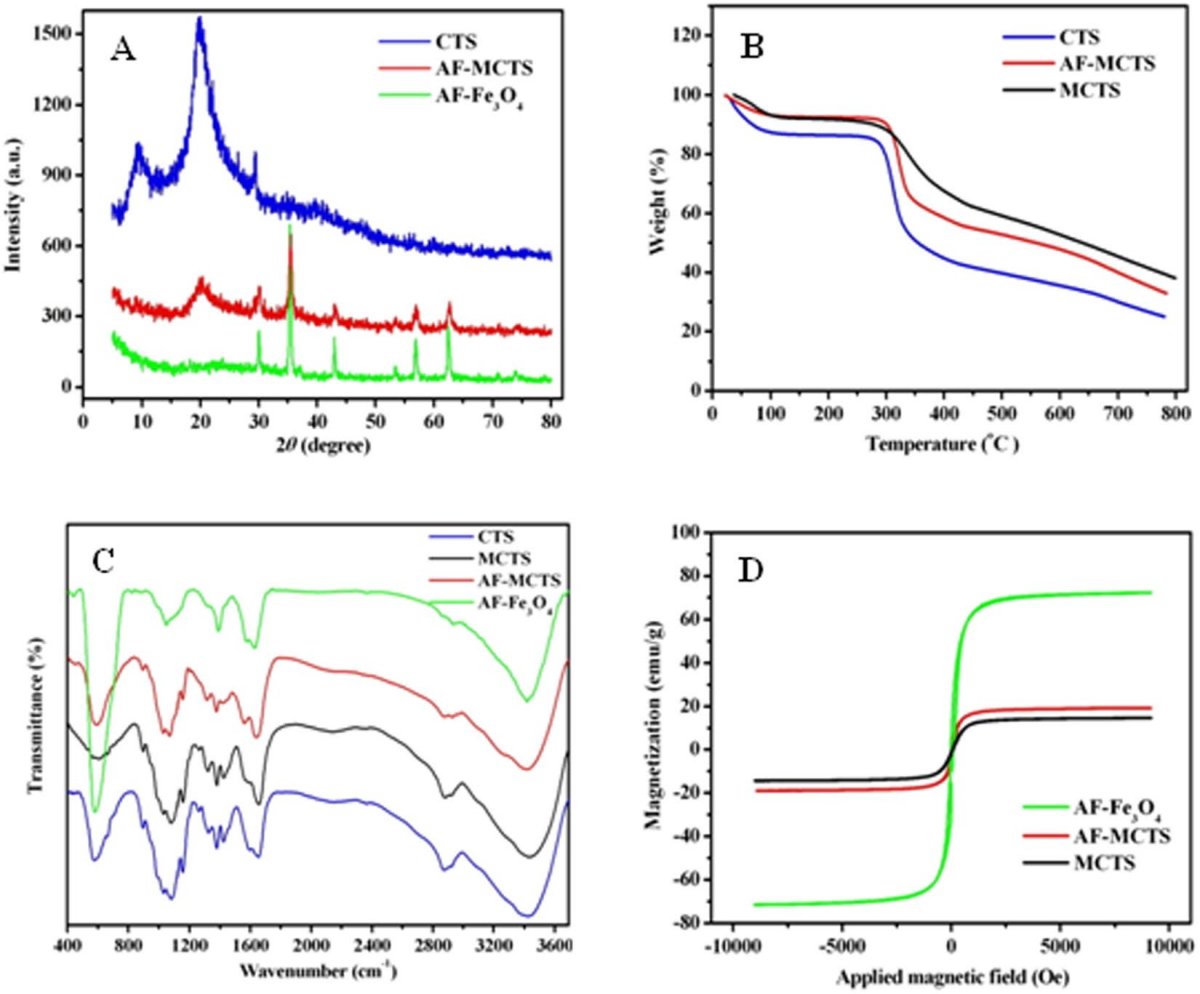
(**A**) XRD patterns of CTS, AF-MCTS, and AF-Fe_3_O_4_. (**B**) TG curves of CTS, MCTS, and AF-MCTS. (**C**) FT-IR spectra of CTS, MCTS, AF-MCTS, and AF-Fe_3_O_4_. (**D**) VSM magnetization curves of MCTS, AF-MCTS, and AF-Fe_3_O_4_.

Figure 2A shows the XRD patterns of CTS, AF-Fe_3_O_4_ and AF-MCTS. The spectrum of CTS showed characteristic peaks located at 10.5° and 20° (2*θ*), which were attributed to (001) and (100), and (101) and (002) planes^31^, respectively. Five characteristic peaks located at 30.1°, 35.5°, 43.3°, 57.2° and 62.5° are attributed to the indices of (220), (311), (400), (511) and (440) of Fe_3_O_4_^32^, which are identified in XRD patterns of AF-Fe_3_O_4_ and AF-MCTS. In addition, it is found that peaks in AF-MCTS are a little weaker than that in AF-Fe_3_O_4_. This is because the conjugation of CTS to Fe_3_O_4_ reduces its crystalline nature to some extent. Meanwhile, it also had a wide diffraction peak at 20°, which was originated from CTS. These observations suggested successful synthesis of magnetic chitosan.

Figure 2B shows the TG data of CTS, MCTS and AF-MCTS. All samples gave a weight loss of 7–13% at 100 °C due to the evaporation of water contained. There was an obvious weight loss occurring at 260–330 °C, which was attributed to decomposition of chitosan chains^33^, following the order of CTS (30%) > AF-MCTS (20%) > MCTS (12%). Compared with MCTS, AF-MCTS gave an extra weight loss (about 8%). This is because synthesis of AF-MCTS was from both CTS and 1,6-hexanediamine, while synthesis of MCTS was from CTS. The weight loss on CTS was higher than that of AF-MCTS and MCTS, indicating that the thermostable Fe_3_O_4_ occupied the part weight of both AF-MCTS and MCTS. Above result suggested the successful preparation of amino-functionalized magnetic chitosan.

Figure 2C displays the infrared spectrum of chitosan, MCTS, AF-MCTS and AF-Fe_3_O_4_. The FT-IR pattern of chitosan showed the main characteristic peaks of chitosan, which were very similar to precious work^15^. For instance, a wide and strong peak at about 3424 cm^−1^ is attributed to OH group of chitosan^15^. The peaks located at 1653 and 3424 cm^−1^ correspond to NH/NH_2_ groups in chitosan^15^. The absorbance bands peaked at 1379 and 2878 cm^−1^ are attributed to CH groups of chitosan, whereas the bands peaked at 1083 and 1031 cm^−1^ are ascribed to C-O groups at C_3_ and C_6_ positions of chitosan^15^, respectively. Compared with CTS, NH_2_ stretching vibration of the AF-MCTS and AF-Fe_3_O_4_ at 3414 cm^−1^ presented a blue shift of 10 cm^−1^. Whereas -NH_2_ bending vibration of the AF-MCTS at 1640 cm^−1^ gave a blue shift of 13 cm^−1^, indicating successful incorporation of 1,6-hexanediamine into MCTS. The characteristic peaks of NH_2_ stretching vibration and -NH_2_ bending vibration of MCTS are almost same as that of CTS. The saturation magnetizations of AF-Fe_3_O_4,_ AF-MCTS and MCTS were 73.18, 19.86 and 15.14 emu/g (Fig. 2D), respectively. It is clear that AF-MCTS and MCTS exhibit significant lower saturation magnetization than AF-Fe_3_O_4_, which was attributed to introducing non-magnetic CTS. However, AF-MCTS and MCTS possess enough high saturation magnetization, which makes AF-MCTS and MCTS much more easy separation from solution by the aid of magnetic field.

### Adsorption ability of the synthesized adsorbents for Cr(VI)

**Figure 3:**
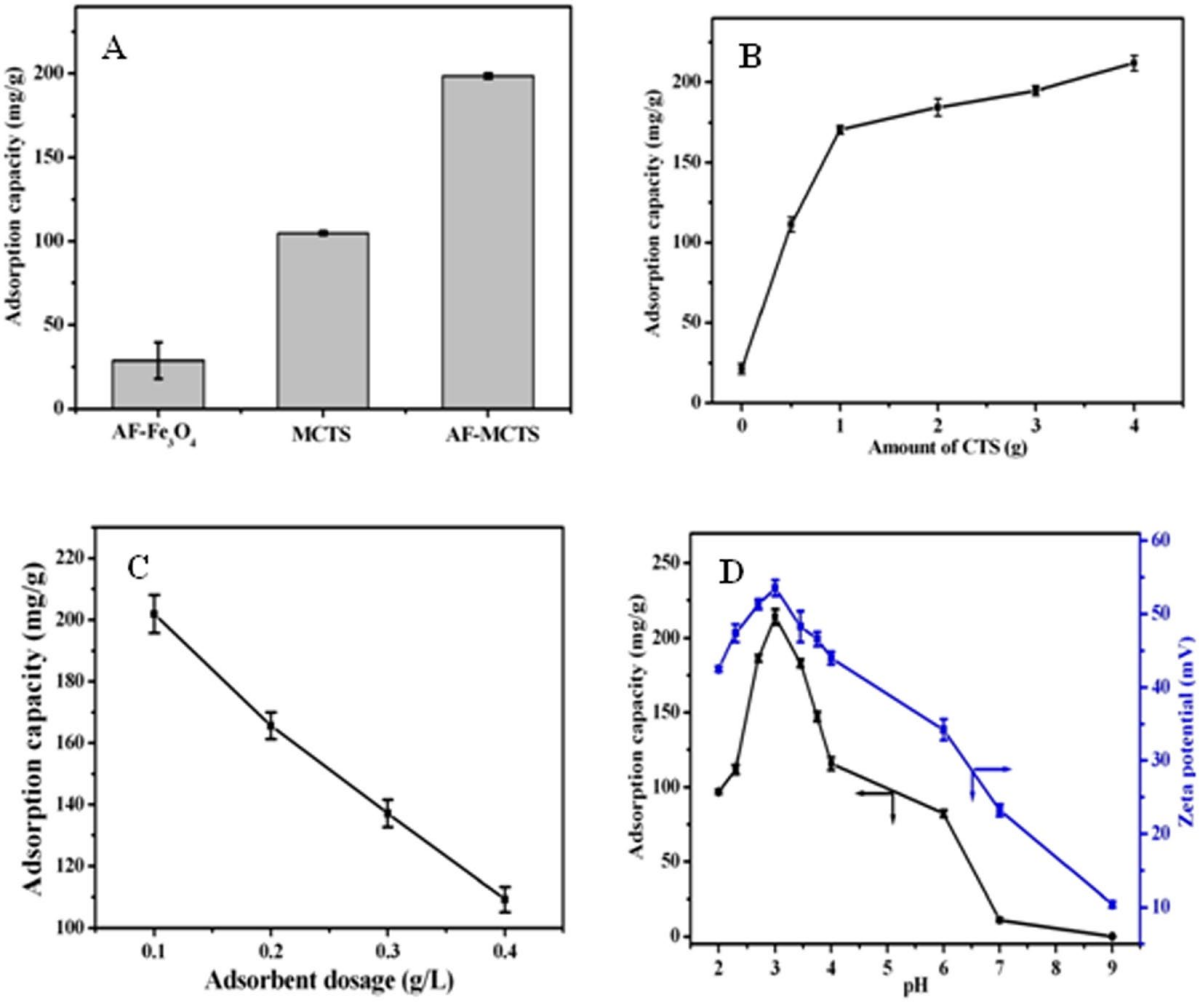
(**A**) Adsorption comparison of AF-Fe_3_O_4_, MCTS, and AF-MCTS for Cr(VI). (**B**) Effect of CTS amount on Cr(VI) adsorption capacity of the resultant AF-MCTS. (**C**) Effect of AF-MCTS-4 dosage on Cr(VI) adsorption. (**D**) Effects of pH on Cr(VI) adsorption and zeta potential of AF-MCTS-4.

Figure 3A shows the adsorption amount of different adsorbents for hexavalent chromium. As seen, the adsorption amount follows the order of AF-MCTS>MCTS>AF-Fe_3_O_4_. The adsorption capacity of AF-MCTS is as high as 200 mg/g, which is nearly twice as that of MCTS, or eight times as that of AF-Fe_3_O_4_ under the identical condition. This implies that Cr(VI) uptake ability by AF-MCTS was remarkably enhanced as compared with MCTS and AF-Fe_3_O_4_, which is probably due to the synergistic effect between CTS and 1,6-hexanediamine. Also, the result implies that the magnetic Fe_3_O_4_ particles play the role of magnetic separation and the adsorption characteristic of the composite is mainly contributed to amino groups and CTS, although the AF-Fe_3_O_4_ has much higher surface area than MCTS and AF-MCTS (Table S2, Supporting Information). This suggests that surface area is not a dominant condition affecting adsorption capacity. This is because 1,6-hexanediamine was used as the functionalized agent having rich -NH_2_ groups, which was favorable for Cr(VI) adsorption. Also, pore volume as well as pore size of AF-MCTS increased compared with that of MCTS after 1,6-hexanediamine modification, thus the external particle surface was accessible for Cr(VI) adsorption. In addition, we prepared ethylenediamine modified MCTS microspheres for hexavalent chromium adsorption. The outcome indicates that ethylenediamine can also be used as the functional reagent to synthesize the amino-functionalized magnetic chitosan microspheres. However, ethylenediamine modified magnetic chitosan microspheres exhibited lower Cr(VI) adsorption capacity than 1,6-hexanediamine modified magnetic chitosan microspheres (Fig. S2, Supporting Information). Hence, in our work, we used 1,6-hexanediamine to prepare the amino-functionalized magnetic chitosan microspheres. In the following work, we systematically studied the adsorption performance of the AF-MCTS for hexavalent chromium as a model contaminant.

### Effect of CTS amount on the adsorption capacity of the AF-MCTS for Cr(VI)

CTS amount influence on adsorption amount of the AF-MCTS for Cr(VI) was explored via varying amount of CTS (0, 0.5, 1.0, 2.0, 3.0, 4.0 g). The usage of CTS have a marked impact on adsorption capacity, which presented in a way that adsorption capacity was rising as increased amount of CTS (Fig. 3B). For example, only about 30 mg/g adsorption capacity was obtained in the absence of CTS. However, the adsorption capacity increases sharply from about 100 mg/g to 175 mg/g when CTS amount increases from 0.5 to 1.0 g, above which it increases slowly. The result indicated the important role of CTS in the Cr(VI) adsorption of the AF-MCTS. In this work, 4.0 g CTS was selected for the preparation of amino-functionalized magnetic chitosan.

### Effect of adsorbent dosage

To do this, in 50 mL Cr(VI) solution (0.1 g/L), the dosage of AF-MCTS-4 adsorbent was varied in the range of 0.1∼0.4 g/L. As shown in Fig. 3C, Cr(VI) adsorption amount on AF-MCTS-4 adsorbent decreased from 202 mg/g to 109 mg/g at AF-MCTS dosage change in the range of 0.1∼0.4 g/L. The possible reason lies that the quantity of available active sites at surface of AF-MCTS-4 increases with rise in its amount used in the experiment. However, some of the adsorption sites remain unsaturated when larger amount of adsorbent was used. This leads to excessive empty adsorption sites in the course of adsorbing Cr(VI), which causes a decrease in Cr(VI) adsorption and waste of adsorbent. Therefore, 0.1 g/L of AF-MCTS-4 was to be optimal dosage for adsorbing Cr(VI).

### Effect of pH

pH effect was studied from pH 2.0 to 9.0. The result in Fig. 3D indicated that pH effect on Cr(VI) adsorption by AF-MCTS-4 was significant, and the removal performance of AF-MCTS-4 for Cr(VI) in acidic condition was better as compared with that in alkaline condition. Upon pH value change from 2 to 3, the adsorption capacity of AF-MCTS-4 for Cr(VI) was enhanced. But the adsorption capacity of AF-MCTS-4 for Cr(VI) declined when the pH value of initial solution increased from 3.0 to 9.0. The highest Cr(VI) adsorption is observed at pH 3.0. The decrease in Cr(VI) adsorption when pH is below 3 may be due to the solubility loss of the adsorbent^28^. The performance of AF-MCTS-4 for Cr(VI) uptake above pH 3.0 is presumably explained based on surface charge of the used AF-MCTS-4 adsorbent and the existence form of Cr(VI) in solution. To this end, we measured the zeta potential of AF-MCTS-4 at different pH from 2.0 to 9.0. As displayed Fig. 3D, the movement trend of zeta potential of AF-MCTS-4 was in accordance with that of adsorption capacity. The speciation of hexavalent chromium in different pH conditions presents mainly five ionic species, namely, H_2_CrO_4_, HCrO_4_^−^, CrO_4_^2−^, HCr_2_O_7_^−^, and Cr_2_O_7_^2−^ ^34^. For a pH above 6.0, CrO_4_^2−^ is a main form of hexavalent chromium, whereas HCrO_4_^−^ and CrO_4_^2−^ are main forms of hexavalent chromium at pH from 2.0 to 6.0. Whereas H_2_CrO_4_ is a major form of hexavalent chromium at pH below 1.0^27^. Therefore, amino groups in both CTS and 1,6-hexanediamine could be protonated to acquire -NH_3_^+^ in acidic medium. And the obtained positively charged -NH_3_^+^ in material surface is in favor of good affinity to negatively charged anionic Cr(VI) through strong electrostatic attraction. Above result suggests that the electrostatic attraction can be regarded as the major mechanism for removing Cr(VI).

### Effect of initial Cr(VI) concentration

**Figure 4:**
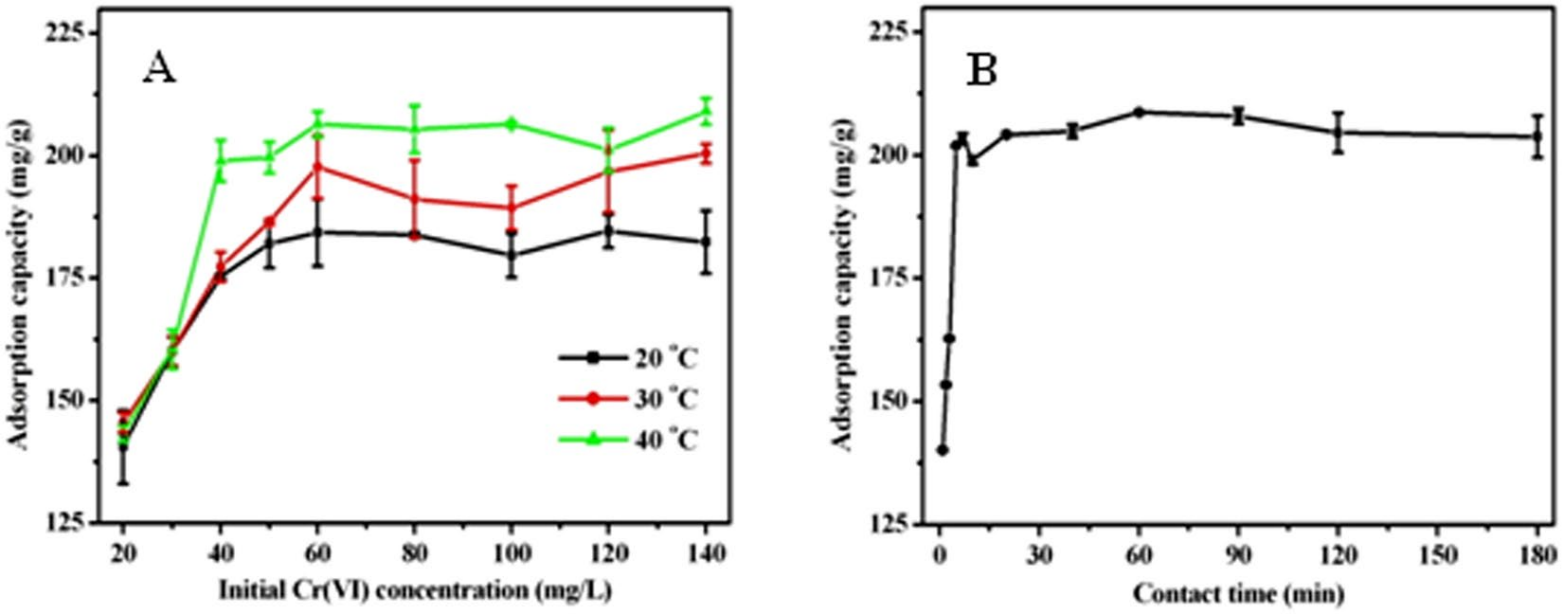
(**A**) Effect of initial Cr(VI) concentration on Cr(VI) adsorption capacity by AF-MCTS-4. (**B**) Effect of contact time on Cr(VI) adsorption capacity by AF-MCTS-4.

As shown in Fig. 4A, the adsorption amount of hexavalent chromium on AF-MCTS-4 was enhanced with the increase of initial hexavalent chromium concentration and achieved a plateau at 60 mg/L level of Cr(VI). The probable reason for the observed result is that there exist large Cr(VI) concentration difference between solution and AF-MCTS-4 surface at high Cr(VI) concentration level, which would lead to intense concentration gradient. This is a major driver of adsorbing Cr(VI), leading to rapid Cr(VI) diffusion to the AF-MCTS-4 surface. A plateau occurs because the adsorption sites of AF-MCTS-4 were saturation. Also, when as solution temperature increased from 20 °C to 40 °C, the adsorption ability of AF-MCTS-4 for hexavalent chromium increased, suggesting that it was an endothermic process for AF-MCTS-4 to adsorb Cr(VI).

### Effect of contact time

Time-dependent adsorption of Cr(VI) by AF-MCTS-4 was explored at Cr(VI) content of 100 mg/L level and pH of 3.0. Cr(VI) uptake by AF-MCTS-4 proceeded promptly in the first 5 min (Fig. 4B), indicating a very fast Cr(VI) adsorption rate exhibited by AF-MCTS-4 adsorbent. This is because an abundant adsorption sites are in favor of Cr(VI) adsorption onto AF-MCTS-4 upon adding AF-MCTS-4 into Cr(VI) solution. However, when the available free surface is saturated by Cr(VI) ions gradually, adsorption process becomes slow and finally reach equilibrium. Also, it is noted that the required time to reach balance for Cr(VI) adsorption onto AF-MCTS-4 was only 5 min for 100 mg/L level Cr(VI) concentration. In our work, a contact time of thirty minutes were selected in the following works to ensure adsorption balance.

### Adsorption kinetic studies

To study adsorption progress, the measured data was modeled by pseudo first-order kinetic formula (eq. 1)^15,18^ and pseudo second-order kinetic formula (eq. 2)^15,18^.

1 $$\mathrm{ln}({q}_{e}-{q}_{t})=\,\mathrm{ln}\,{q}_{e}-{k}_{1}t$$

2 $$\frac{t}{{q}_{t}}=\frac{1}{{k}_{2}{q}_{e}^{2}}+\frac{t}{{q}_{e}}$$

In above equations, *q*_*e*_ and *q*_*t*_ denote adsorption amount (mg/g) at equalization and time *t* (min), respectively. *k*_1_ and *k*_2_ represent the first and second order kinetic constants, respectively.

The kinetic parameters and correlation coefficient (*r*^2^) were acquired by linear fitting the experimental data (Table 1). The experimental data matched much better in pseudo-second-order formula than in pseudo-first-order formula in terms of values of correlation coefficient. For Cr(VI) adsorption by AF-MCTS-4, the estimated adsorption amount by the pseudo-second-order formula was 204.08 mg/g. Whereas it was 203.84 mg/g in terms of the measurement data (Table 1). Hence, the calculated theoretical *q*_*e*_ in terms of pseudo-second-order equation is highly approaching to the measured *q*_*e*_. Thus, the overall course for adsorbing Cr(VI) by AF-MCTS material followed the pseudo-second-order equation.

**Table 1:** Kinetic parameters of pseudo-first-order and pseudo-second-order models for Cr(VI) adsorption by AF-MCTS.

Pseudo-first-order model				Pseudo-second-order model		
*q*_*exp*_ (mg/g)	*q*_*e*_ (mg/g)	*k*_1_ (min^−1^)	*r* ^2^	*q*_*e*_ (mg/g)	*k*_2_ (g/mg min)	*r* ^2^
203.84	69.92	0.4443	0.5151	204.08	0.040	0.9999

### Adsorption isotherms

The Langmuir and Freundlich were applied to fit the measured data obtained from the process of adsorbing different Cr(VI) concentration levels by AF-MCTS. The former assumes a homogeneous adsorption process while the latter describes heterogeneous systems, which can be expressed by the following equation 3^15,16^ and equation 4^15,16^, respectively.

3 $$\frac{{C}_{e}}{{q}_{e}}=\frac{{C}_{e}}{{q}_{\max }}+\frac{1}{{q}_{\max }b}$$

4 $$\mathrm{lg}\,{q}_{e}=\,\mathrm{lg}\,k+\frac{1}{n}\,\mathrm{lg}\,{C}_{e}$$

where *C*_*e*_ (mg/L) represents content of hexavalent chromium when the adsorption reaches equilibrium. *q*_*e*_ (mg/g) represents the quantity of hexavalent chromium adsorbed at adsorption balance. *b* represents Langmuir factor (L/mg) which is associated with adsorption energy, while *q*_*max*_ (mg/g) represents the maximal monolayer adsorption amount. *k* and *n* represent Freundlich factors, which are associated with adsorption capacity and adsorption intensity, respectively.

The linear fitting curves for hexavalent chromium adsorption by AF-MCTS obtained according to Langmuir and Freundlich models are displayed in Fig. S3A,B (Supporting Information), respectively. The corresponding isotherm parameters obtained by the two models were given in Table 2. The result indicates the suitability of Langmuir model to describe the isothermal adsorption of Cr(VI) by AF-MCTS-4, suggesting the monolayer coverage of Cr(VI) ions onto AF-MCTS-4. On the basis of Langmuir isothermal curve, the maximal Cr(VI) uptake amount is 208.33 mg/g, which is 1.1 to 12 times that of other CTS-based adsorbents (Table 3)^13,15–20,22–26,28,29,31,35–39^. Furthermore, the uptake rate of hexavalent chromium by AF-MCTS-4 was very fast. Only 5 min was needed to reach adsorption equilibrium, which was much faster than other CTS-based adsorbents (Table 3). Hence, the AF-MCTS-4 performs better than most other CTS-based adsorbents in terms of adsorption capacity and in particular adsorption time.

**Table 2:** Parameters obtained by the Langmuir and Freundlich models for Cr(VI) adsorption onto AF-MCTS.

Langmuir model			Freundlich model		
*q*_*max*_ (mg/g)	*b* (L/mg)	*r* ^2^	*k* (mg/g)	*n*	*r* ^2^
208.33	0.6153	0.9989	137.12	10.5374	0.8165

**Table 3:** Comparison of Cr(VI) adsorption performance by AF-MCTS with other chitosan-based adsorbents.

Adsorbents	Q_max_ (mg/g)	Time (min)	Refs.
Chitosan	35.6	120	^13^
Titanium cross-linked chitosan composite	171	420	^15^
Zirconium cross-linked chitosan	175	300	^16^
Chitosan–Fe(III) complex	173.1	10	^17^
Ethylenediamine cross-linked magnetic	51.8	6–10	^18^
Chitosan resin			
Ethylenediamine modified chitosan	60.9	120	^19^
*n*-butylacrylate grafted chitosan	17.15	60	^20^
CTS/montmorillonite-Fe_3_O_4_	35.71–58.82	90	^22^
Magnetic cyclodextrin-chitosan/graphene oxide	67.66	300	^23^
Chitosan-coated MnFe_2_O_4_ nanoparticles	35.32	360	^24^
Cross-linked magnetic chitosan beads	69.4	60	^25^
Magnetic chitosan nanoparticles	55.8	100	^26^
Magnetic chitosan–GO nanocomposite	101.6	120	^28^
CTS-iron(III) hydrogel	144.9	30	^29^
Modified magnetic chitosan chelating resin	58.5	120	^31^
Cross-linked chitosan resin	86.8–112.7	120	^35^
Chitosan modified Fe^0^ nanowires	113.2	400	^36^
Poly(ethylene imine) grafted chitosan	88.4	105	^37^
Pyridinium functionalized magnetic chitosan	175.94	120	^38^
Chitosan crosslinked with diethylenetriaminepentaacetic acid	192.3	240	^39^
Amino-functionalized magnetic chitosan	208.33	5	present work

### Effect of the ionic strength

**Figure 5:**
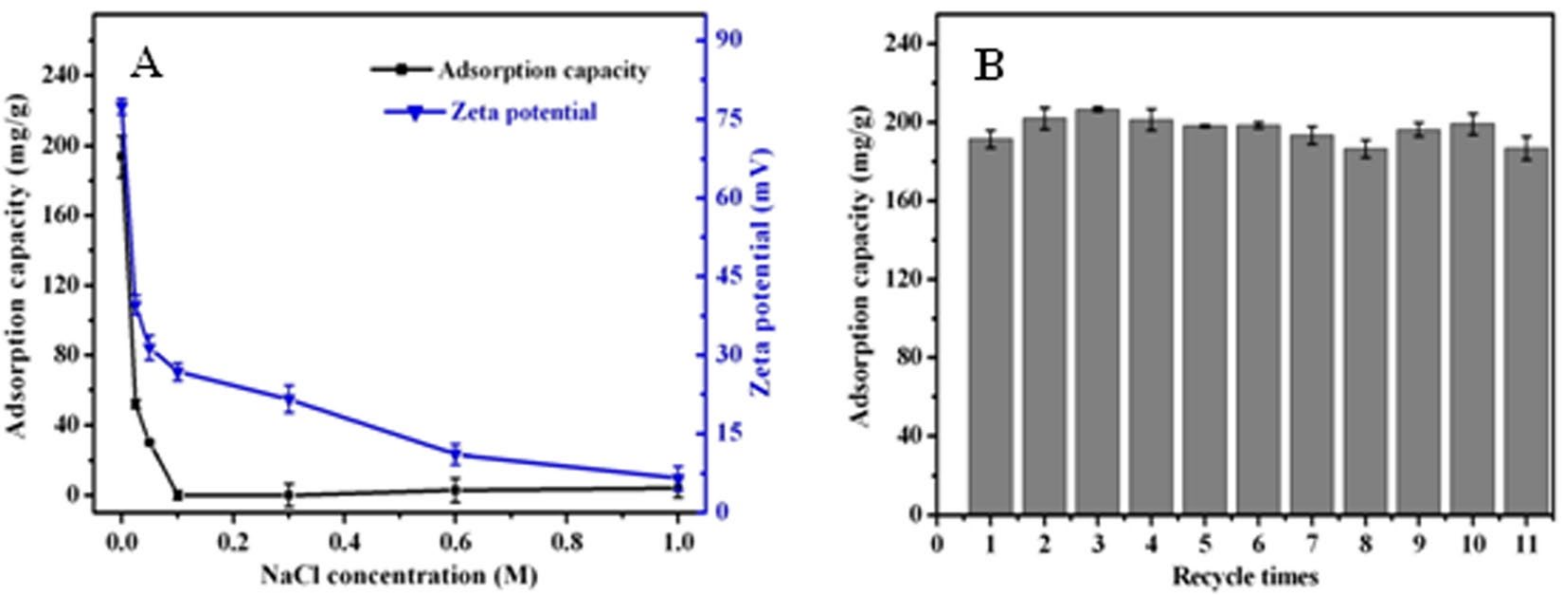
(**A**) Effect of ion strength on Cr(VI) adsorption by AF-MCTS-4 and the zeta potential of the AF-MCTS-4. (**B**) Variation of adsorption capacity of AF-MCTS-4 for Cr(VI) with recycle runs under the same experimental conditions.

Figure 5A displays the result of ionic strength effect on Cr(VI) adsorption by AF-MCTS in the presence of NaCl. Cr(VI) adsorption on AF-MCTS decreases sharply with increase in concentration of sodium chloride from 0 to 0.1 M, then remains slight change when NaCl concentration was >0.1 M. For example, the Cr(VI) adsorption amount declined from 193.58 mg/g to 51.51 mg/g when NaCl concentration ranged from 0 to 0.025 M. However, when NaCl concentration reached 0.1 M, there is almost no retention of Cr(VI) on AF-MCTS. This indicates that electrostatic interaction mechanism plays a crucial part in hexavalent chromium uptake by AF-MCTS. To confirm this, we measured the zeta potential variances of AF-MCTS in the presence of different concentrations of NaCl (Fig. 5A). It is clear that addition of NaCl leads to a big decline in zeta potential value of AF-MCTS, suggesting that addition of Cl^−^ ions results in the decrease in the available surface sites for hexavalent chromium adsorption. This is because the occupied sites decrease surface charge of AF-MCTS adsorbent, leading to increase in the electrostatic repulsion between AF-MCTS surface and Cr(VI) anions.

### Adsorption thermodynamic study

The free energy change (∆*G*), enthalpy change (∆*H*) and entropy change (∆*S*) were estimated based on subsequent three formulas:

5 $$\mathrm{ln}({K}_{d})=\frac{{\rm{\Delta }}S}{R}-\frac{{\rm{\Delta }}H}{RT}$$

6 $${\rm{\Delta }}G={\rm{\Delta }}H-T{\rm{\Delta }}S$$

7 $${K}_{d}=\frac{{q}_{e}}{{C}_{e}}$$

where *K*_*d*_ represents distribution coefficient at varied temperature, *q*_*e*_ and *C*_*e*_ represent content of hexavalent chromium adsorbed by the used material (mg/g) and in aqueous phase (mg/L), *R* is gas constant, *T* represents absolute temperature (*K*).

Values of three thermodynamic parameters were exhibited in Table S3. ∆*G* value is less than zero, suggesting the spontaneous feature of Cr(VI) adsorption by AF-MCTS. In addition, with temperature increasing, the absolute value of ∆*G* increased obviously, indicating that Cr(VI) adsorption by AF-MCTS is easy to proceed at higher temperature. ∆*H* value is above zero, confirming that Cr(VI) adsorption by AF-MCTS is the endoergic process, which agrees with the result of temperature effect on Cr(VI) uptake. Similar to ∆*H* value, ∆*S* value is also greater than zero, suggesting that the randomness in solid/liquid interphase would be enhanced in the course of Cr(VI) adsorption by AF-MCTS.

### Recyclability of AF-MCTS for Cr(VI) removal

To explore recyclability of AF-MCTS adsorbent, the regeneration and cyclic use of the adsorbent were conducted. To this end, after being treated by Cr(VI) solution for 30 min, the AF-MCTS adsorbent was regenerated with 10 mL of 0.5 M NaOH. The generated AF-MCTS adsorbent was then used for the subsequent uptake of Cr(VI) under the identical experimental conditions. As shown in Fig. 5B, after the first use, about 97% Cr(VI) removal was remained even after eleven cycles, suggesting that the AF-MCTS adsorbent exhibited no obvious decline in Cr(VI) uptake over adsorption eleven cycles. Therefore, the AF-MCTS material exhibits favorable recyclability.

### Possible mechanism of Cr(VI) removal

**Figure 6:**
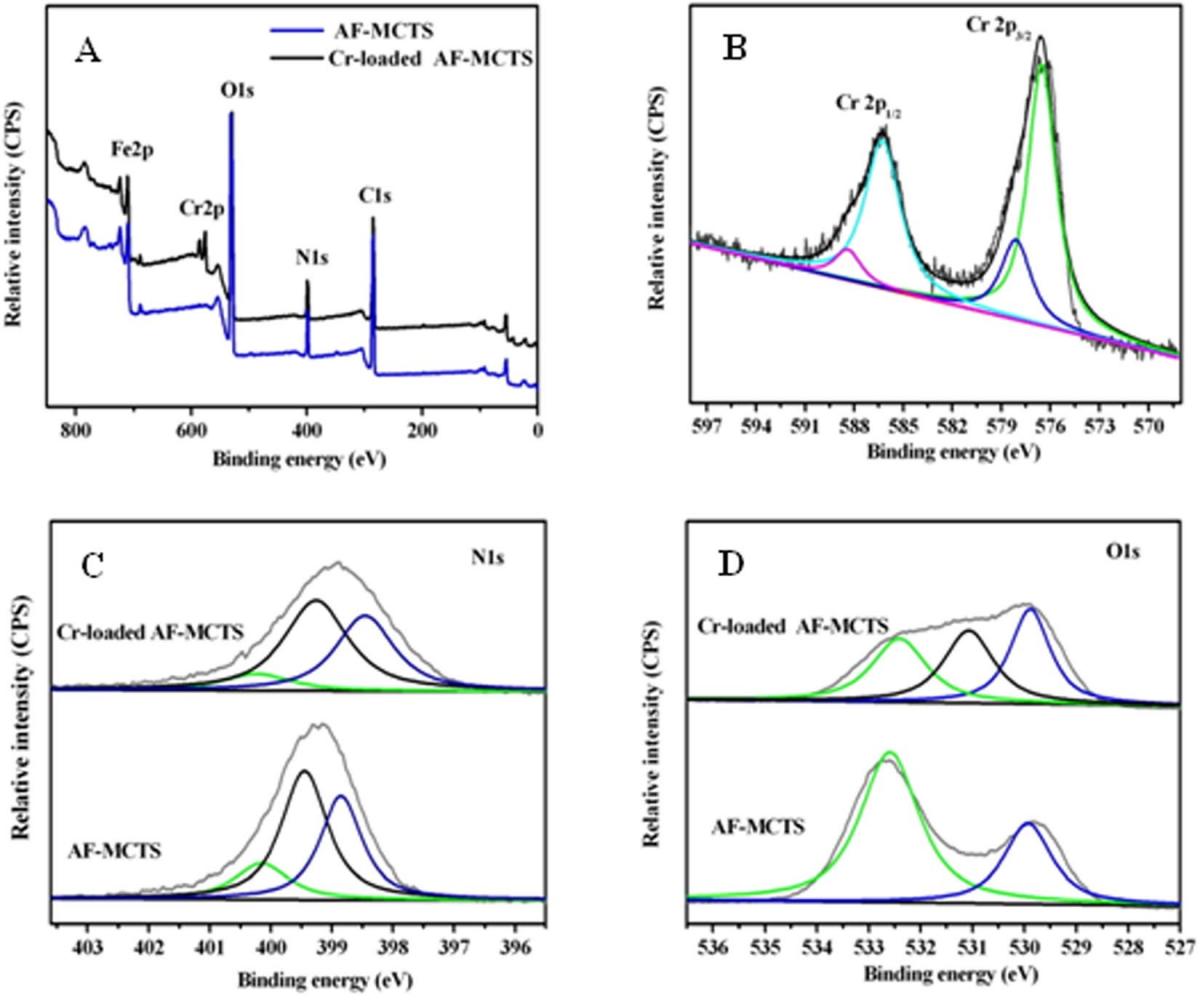
(**A**) XPS survey spectra of AF-MCTS and Cr-loaded AF-MCTS. (**B**) XPS spectra of AF-MCTS in the Cr2p region after Cr(VI) treatment. (**C**) XPS spectra in the N1s region of AF-MCTS and Cr-loaded AF-MCTS. (**D**) XPS spectra in the O1s region of AF-MCTS and Cr-loaded AF-MCTS.

To look into possible mechanism for Cr(VI) removal by AF-MCTS, an XPS characterization was carried out on AF-MCTS materials with and without treatment by Cr(VI) solution to gain insight into the variance of chemical component on AF-MCTS surface, and the result of measurements in XPS survey spectra was given in Fig. 6A. From the element analysis, chromium has been successfully adsorbed by AF-MCTS after Cr(VI) treatment. There are two peaks at 576.7 eV (attributed to Cr2p_3/2_ line) and at 586.3 eV (attributed to Cr2p_1/2_ line) in the high resolution Cr2p spectra (Fig. 6B), respectively. Two peaks at 576.3 and 578.18 eV deconvoluted from Cr2p_3/2_ are features of Cr^3+^ and Cr(VI)^17^, respectively. This indicates that Cr(VI) and Cr^3+^ are present on the AF-MCTS adsorbent after Cr(VI) adsorption. The presence of Cr(VI) on the surface of AF-MCTS is attributed to its uptake by AF-MCTS through electrostatic interaction. From the result of zeta potential research (Fig. 3D), the functional groups on AF-MCTS composite could be positively charged (especially amino groups could be protonated into positively charged –NH_2_^+^/-NH_3_^+^) in acidic medium. The positively charged surface of AF-MCTS composite had a strong electrostatic affinity to anionic Cr(VI) (HCrO_4_^−^). The electrostatic interaction is demonstrated by the result of ionic strength influence (Fig. 5A). In addition, the existence of Cr^3+^ on AF-MCTS adsorbent suggests partial reduction of Cr(VI) retained by AF-MCTS to Cr^3+^ through chemical reducing interaction. Interestingly, no detectable Cr^3+^ ions were found in the treated water solution after reaction between Cr(VI) and AF-MCTS at different initial solution pH values (Fig. S4, Supporting Information). This suggests that Cr(III) formed during reduction of hexavalent chromium is also removed by AF-MCTS. In order to gain insight into the possible electron donor involved in reducing hexavalent chromium and the probable reason for the existence of Cr^3+^ in AF-MCTS, XPS spectra in the N1s region and O1s region were analyzed. From the N1s spectra of the as-prepared AF-MCTS (Fig. 6C), the binding energies located at 400.2, 399.5 and 398.8 eV are assigned to N atoms in -NH_2_^+^/-NH_3_^+^, -NH/-NH_2_ and -NH_2_ interacted with Fe(III)^17^, respectively. This is because the -NH and -NH_2_ groups in chitosan chains were protonated into -NH_2_^+^/-NH_3_^+^. The formed Cr^3+^ in the course of hexavalent chromium reduction was allowed to coordinate with -OH groups in chitosan chains^40^. At the same time, the proton depletion due to Cr(VI) reduction caused deprotonation of -NH_2_^+^/-NH_3_^+^ to -NH and -NH_2_ groups. Hence, the formed Cr^3+^ was also admitted to coordinate with neighboring groups such as -NH_2_ and -NH groups^41^ through supramolecular coordination^42^ by sharing electrons with the N atom in these groups, leading to a shift to lower binding energy of N1s after Cr(VI) adsorption because of increase in the electron cloud density of nitrogen atom caused by deprotonation of -NH_2_^+^/-NH_3_^+^. However, when the AF-MCTS was regenerated by sodium hydroxide and subsequently used for next cycle, the supramolecular coordination between -NH_2_ and –NH groups in chitosan and Cr^3+^ was cleaved to release -NH_2_ and -NH groups^42^, which were again protonated into -NH_2_^+^/-NH_3_^+^ at pH 3.0 acidic solutions, and to facilitate re-coordination of Cr^3+^ and -OH groups in chitosan (Fig. S5, Supporting Information), leading to accumulation of Cr(III) in the AF-MCTS (Fig. S6, Supporting Information). Thus, consumption or addition H^+^ could trigger the reversible supramolecular coordination between Cr^3+^ and chitosan. Hence, the -NH_2_ groups in AF-MCTS is not the electron donor for Cr(VI) reduction, which was in consistent with result reported by Shen *et al*.^17^. On the other hand, for AF-MCTS, the dominant peak in O1s region (Fig. 6D) at 530.0 eV corresponds to Fe-O^43^ structure in metal oxides. Whereas another peak at about 532.6 eV is attributed to -OH oxygen in chitosan^44^. Interestingly, being treated by hexavalent chromium solution, the intensity of hydroxyl peak located at 532.6 eV is significantly declined (Fig. 6D). At the same time, the binding energy of O1s in AF-MCTS-4 shifts to a lower value of 532.05 eV, indicating contribution of hydroxyl groups to adsorbing hexavalent chromium. Also, a new peak appears at 530.7 eV, which is the characteristic peak of C=O derived from oxidation of C-OH by hexavalent chromium^15,17^. This result suggests that hydroxyl groups in AF-MCTS were the electron donors for reducing hexavalent chromium. Similar result was also found in hexavalent chromium reduction by CTS–Fe(III)^17^. It is noteworthy that presence of -OH groups can be proved by characteristical peak located at 1083 cm^−1^ in FT-IR spectra of AF-MCTS treated by Cr(VI) after repeated use (Fig. S7, Supporting Information). Hence, the AF-MCTS could maintain the high adsorption capacity during the reuse. In summary, the -OH groups rather than –NH_2_ groups in the AF-MCTS were the electron donor for reducing Cr(VI). The AF-MCTS adsorbent could maintain high adsorption capacity mainly as result of electrostatic attraction between protonated -NH_2_^+^/-NH_3_^+^ and anionic Cr(VI) in acidic medium during the reuse.

**Figure 7:**
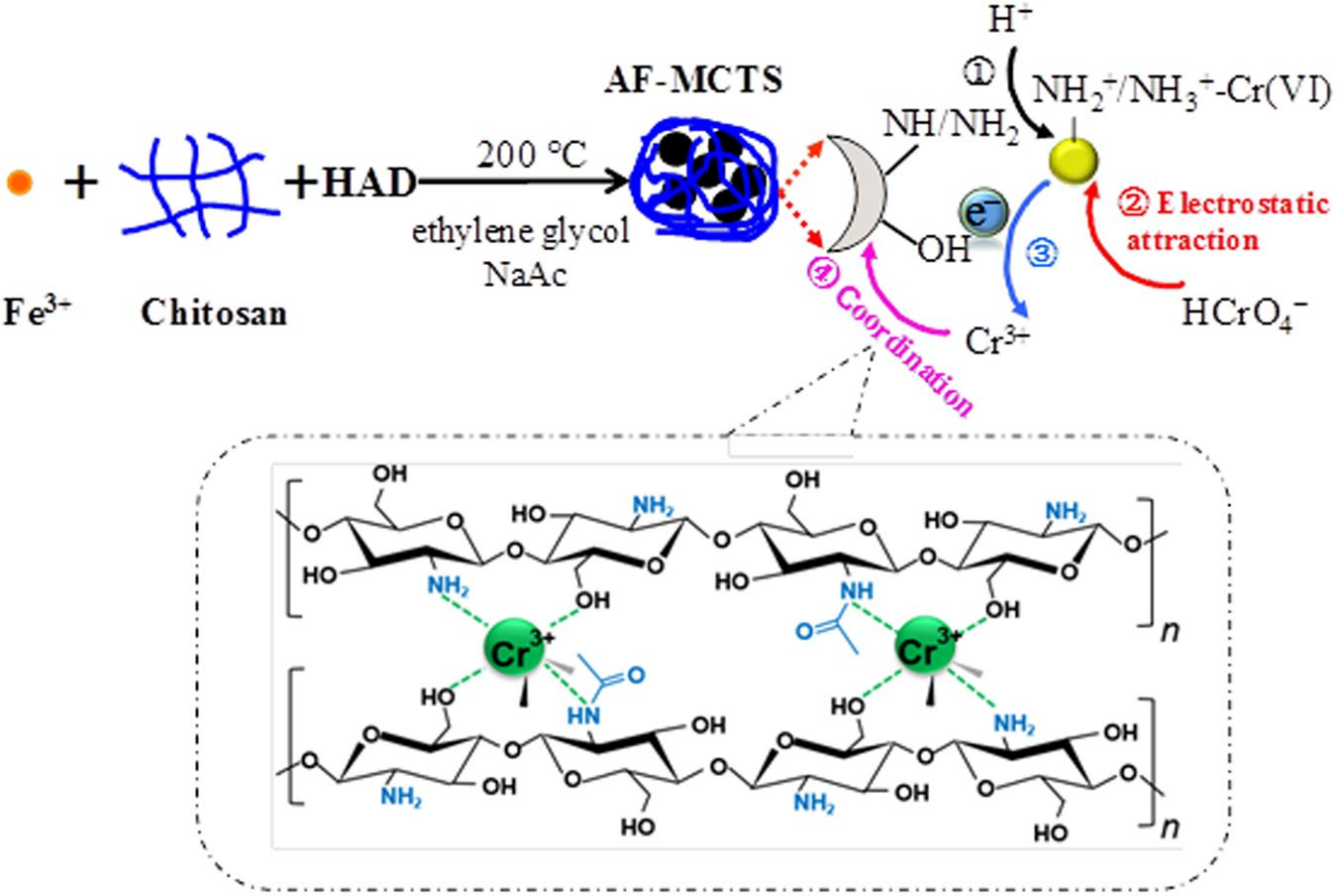
The proposed removal mechanism of Cr(VI) by AF-MCTS.

On above discussion basis, a probable mechanism for the removal of Cr(VI) with AF-MCTS adsorbent is proposed, and it contains two main steps (Fig. 7): (1) protonation of the -NH and -NH_2_ groups in chitosan chains into -NH_2_^+^/-NH_3_^+^ at pH 3.0 media, leading to Cr(VI) diffusion from aqueous solution to positive charged AF-MCTS adsorbent through electrostatic interaction; (2) partial hexavalent chromium reducing to Cr^3+^ by -OH groups onto AF-MCTS, and the formed Cr^3+^ was re-adsorbed by the AF-MCTS through supramolecular coordination.

## Conclusion

In summary, we demonstrated the development of AF-MCTS for rapid and efficient elimination of toxic Cr(VI) from aqueous solution through adsorption. Compared with other CTS-based adsorbents, AF-MCTS exhibited unique characteristics as follows: (1) it can be prepared by one-step hydrothermal route without the requirement of the complicated multi steps. (2) Importantly, adopting one-step hydrothermal route successfully overwhelms serious drawbacks of using cross-linking reaction for grafting chitosan onto magnetic materials, such as consumption of amino groups in CTS and low Cr(VI) uptake ability of the resultant adsorbents. (3) The size of the resultant adsorbents is easily regulated by directly governing the amount of amino-functionalized agent and CTS, which is very important for obtaining adsorbent with very fast Cr(VI) adsorption ability. (4) The AF-MCTS could be repeatedly utilized by simple regeneration through NaOH. These advantages make the one-step hydrothermal strategy attractive for future construction of high performance materials for removal of pollutants through adsorption.

### Experimental section

#### Reagents and chemicals

The chemical substances purchased are at least analytical grade and are employed with no further treatment. Chitosan (The average molecular weight is 300000 g/moL based on viscosity, with deacetylation degree of 85%.) was purchased from Nantong Xingchen Biological Co. Ltd. (Nantong, Jiangsu). 1,6-Hexanediamine was provided by Aladdin (Shanghai, China). FeCl_3_·6H_2_O and sodium acetate (NaAc) were provided by Kelong Chemical Reagent Co. Ltd. (Chengdu, Sichuan). The other chemicals such as NaOH, ethylene glycol, acetone, ethanol, and hydrochloric acid were purchased from Chongqing Taixin Chemical Co. Ltd. (Beibei, Chongqing).

#### Preparation of AF-MCTS

AF-MCTS was synthesized by a single-step hydrothermal procedure. In detail, 2 g ferric chloride was added into 60 mL glycol, followed by addition of 4 g NaAc and 10 g 1,6-hexanediamine successively. Then, the different amount of 0.5~4 g CTS powders were added into to obtain a mixture solution, which was agitated continuously for 12 h, then transferred into Teflon lined stainless-steel autoclave, which was sealed and heated to 200 °C. After heating to 200 °C and keeping at this temperature for 8 h, the reaction was finished. The solid product was washed by C_2_H_5_OH, H_2_O in order, dried at 60 °C for a day. Based on the amount of CTS (0, 0.5, 1.0, 2.0, 3.0, 4.0 g) used in this work, the resultant products are denoted as AF-MCTS-0.5, AF-MCTS-1, AF-MCTS-2, AF-MCTS-3, and AF-MCTS-4, respectively. The dried composite was used for characterizations and adsorbing Cr(VI) studies. Furthermore, for the purpose of comparison, we prepared amino-functionalized Fe_3_O_4_ (AF-Fe_3_O_4_) and magnetic chitosan (MCTS) using the same method without adding CTS and 1,6-hexanediamine, respectively.

#### Characterization and measurement

Thermogravimetric (TG) measurements were carried out by a TA-SDTQ 600 thermal analyzer (Texas Instruments, USA), which was heated at 10 °C per minute from 35 °C up to 800 °C. The powder sample was analyzed by the PuXi XD-3 XRD (Peking, China) to obtain diffraction characteristic of the synthesized materials. The Fourier Transform Infrared spectrometer (Tenson 27, Bruker, Germany) was used to record the FT-IR spectra of materials. The TEM analysis was carried out by using a Tecnai G2-20 transmission electron microscope (FEI, USA). An energy dispersive spectroscopy (EDS) of the sample was obtained by using a scanning electron microscope (Merlin Compact, Zeiss, Germany). The nitrogen adsorption–desorption isotherms of as-prepared adsorbents were determined on an ASAP 2020 Micromeritics instrument (Maike, USA) at 77 K. The specific surface areas were calculated based on the Brunauer–Emmett–Teller (BET) method and the pore size distribution curve was acquired based on the Barrett–Joyner–Halenda (BJH) model. The zeta potential of the sample was determined by a Zetasizer Nano-ZS Instruments (Malvern, UK.). A U-4100 spectrophotometer (Hitachi, Japan) was used for measuring absorbance.

#### Adsorption of Cr(VI) by the adsorbent

Batch experiment was designed to study adsorption capacities of AF-MCTS hybrid for hexavalent chromium. To this end, 50 mL hexavalent chromium solution and 5 mg various materials (AF-MCTS, AF-Fe_3_O_4_ and MCTS) were placed into a round of 100 mL conical flasks. The mixture was agitated at 180 rpm in a manner of cyclotron oscillation for a given period of time. The Cr(VI) adsorption amount of AF-MCTS was calculated based on the following equation (Eq. 8):

8 $${q}_{e}=\frac{({C}_{0}-{C}_{e})V}{m}$$

where *C*_0_ and *C*_e_ (mg L^−1^) are the initial and equilibrium concentration of Cr(VI), respectively. *V* (L) is the volume of Cr(VI) solution, and *m* (g) is the mass of AF-MCTS. On completion of adsorption, the mixture was filtered by a 0.22 µm membrane. The spectrophotometry was adopted to determine Cr(VI) content in the resultant filtrate.

For the study of adsorption kinetic, the adsorption was carried out by addition of 5 mg AF-MCTS into 50 mL of 10 mg/L Cr(VI) solution (pH 3.0) at 30 °C. At different time interval in the range of 0–180 min, Cr(VI) adsorption capacity was obtained according to Eq. 1. For the study of adsorption isotherms, the adsorption was carried out by addition of 5 mg AF-MCTS into 50 mL of Cr(VI) solution (pH 3.0) with different concentrations from 20 to 140 mg/L. After 30 min adsorption at 30 °C, Cr(VI) adsorption capacity was obtained according to Eq. 8.

## Electronic supplementary material

Supplementary Information
