# Supplementary material for: One-step synthesis of 1,6-hexanediamine modified magnetic chitosan microspheres for fast and efficient removal of toxic hexavalent chromium
Source: Sci Rep. 2018 Jul 23;8:11024. doi: 10.1038/s41598-018-29499-z (PMC6056533; doi:10.1038/s41598-018-29499-z)
Supplement: Supplementary file 1 — Supplementary Information [file 41598_2018_29499_MOESM1_ESM.doc]

**Supporting Information**

**One-step synthesis of 1,6-hexanediamine modified magnetic chitosan microspheres for fast and efficient removal of toxic hexavalent chromium**

Rui Yue, Qiumeng Chen, Siqi Li, Xiaodan Zhang, Yuming Huang,* and Ping Feng*

Key Laboratory of Eco-environments in Three Gorges Reservoir Region (Ministry of Education), College of Chemistry and Chemical Engineering, Southwest University, Chongqing 400715, China.

*Corresponding authors. Tel./Fax: +86-23-68254843 (Y. Huang), +86-23-68254346 (P. Feng); E-mail addresses: yuminghuang2000@yahoo.com (Y. Huang), fenging@swu.edu.cn (P. Feng)

**Table S1.** Comparison of the AF-MCTS with other chitosan-based adsorbents for Cr(VI) adsorption

| Adsorbents | Q_max_  (mg/g) | Time (min) | Cross-linking agents | Steps | Ref. |
| --- | --- | --- | --- | --- | --- |
| Chitosan | 35.6 | 120 | / |  | 13 |
| Titanium cross-linked chitosan composite | 171 | 420 | glutaraldehyde | Two | 15 |
| Zirconium cross-linked chitosan | 175 | 300 | glutaraldehyde | Two | 16 |
| Chitosan–Fe(III) complex | 173.1 | 10 | glutaraldehyde | Two | 17 |
| Ethylenediamine cross-linked magnetic CTS resin | 51.8 | 6−10 | glutaraldehyde | Three | 18 |
| n-butylacrylate grafted chitosan | 17.15 | 60 | / |  | 20 |
| CTS/montmorillonite-Fe_3_O_4_ | 35.71−58.82 | 90 | glutaraldehyde | Three | 22 |
| Magnetic cyclodextrin-chitosan/graphene oxide | 67.66 | 300 | glutaraldehyde | Three | 23 |
| Chitosan-coated MnFe_2_O_4_ nanoparticles | 35.32 | 360 | / | Two | 24 |
| Cross-linked magnetic chitosan beads | 69.4 | 60 | epichlorohydrin | Three | 25 |
| Magnetic chitosan nanoparticles | 55.8 | 100 | epichlorohydrin | Three | 26 |
| Magnetic chitosan–GO nanocomposite | 101.6 | 120 | / | Three | 28 |
| CTS-iron(III) hydrogel | 144.9 | 30 | glutaraldehyde | Three | 29 |
| Modified magnetic chitosan chelating resin | 58.5 | 120 | glutaraldehyde | Three | 31 |
| Cross-linked chitosan resin | 86.8−112.7 | 120 | epichlorohydrin | Two | 35 |
| Chitosan modified Fe^0^ nanowires | 113.2 | 400 | / | Two | 36 |
| Poly(ethylene imine) grafted chitosan | 88.4 | 105 | glutaraldehyde |  | 37 |
| Amino-functionalized magnetic chitosan | 208.33 | 5 | / | One | this work |

**Table S2**. The surface area, total pore volume and total adsorption average pore width of MCTS, AF-Fe_3_O_4_ and AF-MCTS-4

| Adsorbents | Surface area (m^2^/g) | Total pore volume (cm^3^/g) | Total adsorption average pore width (nm) |
| --- | --- | --- | --- |
| MCTS | 5.30 | 0.02 | 14.66 |
| AF-Fe_3_O_4_ | 35.45 | 0.21 | 23.45 |
| AF-MCTS-4 | 6.22 | 0.05 | 34.08 |

**Table S3**. Thermodynamic parameters for Cr(VI) adsorption by the AF-MCTS-4 composite at different temperatures.

| *T* (*K*) | Ln(*K*_d_) | ∆*G* (kJ/mol) | ∆*H* (kJ/mol) | ∆*S* (kJ/mol) | *r*^2^ |
| --- | --- | --- | --- | --- | --- |
| 293 | 1.501 | -3.656 | 3.5007 | 0.0245 | 0.9974 |
| 303 | 1.557 | -3.922 |  |  |  |
| 313 | 1.593 | -4.145 |  |  |  |


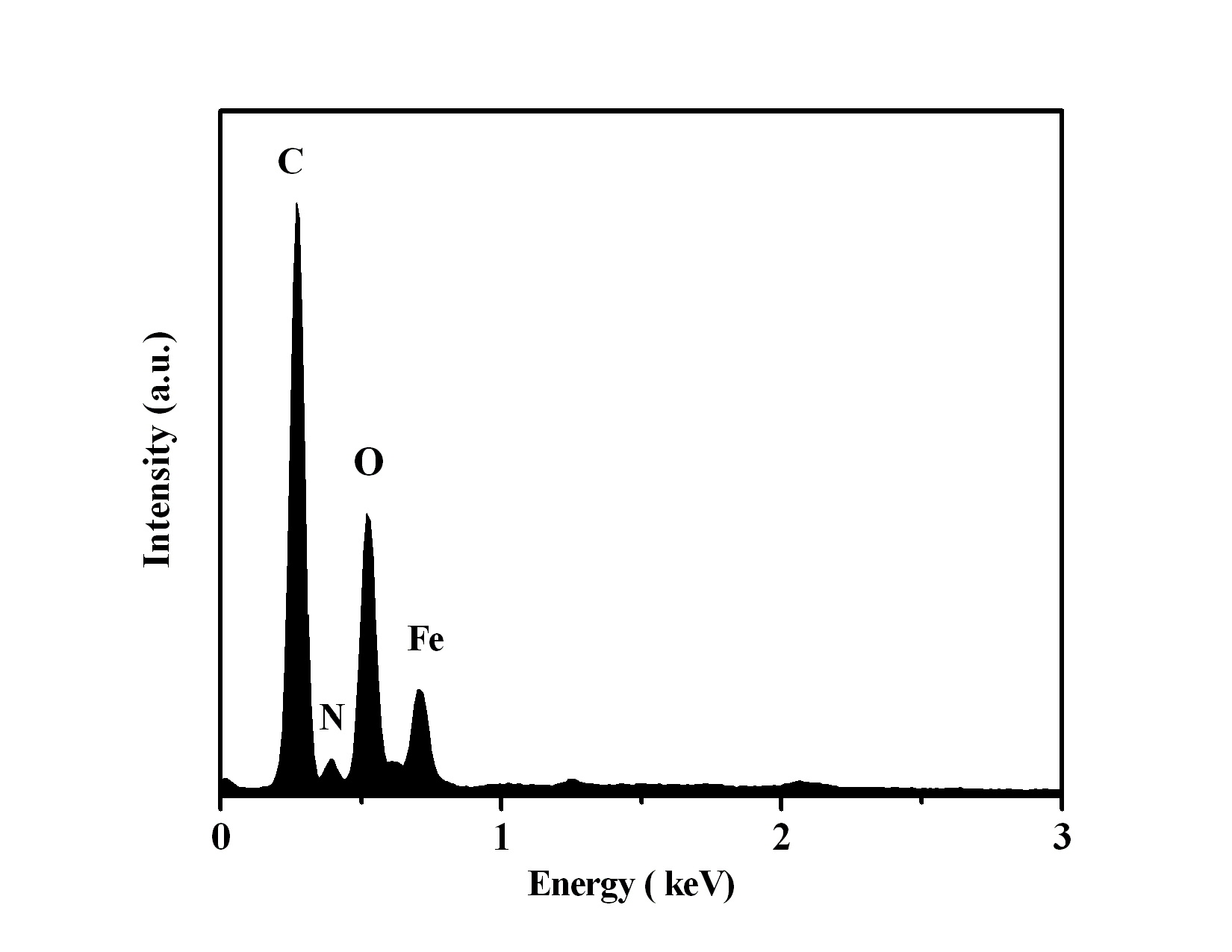


| Element | Atomic% |
| --- | --- |
| *CK* | 62.42 |
| *NK* | 7.99 |
| *OK* | 26.16 |
| *FeK* | 3.43 |

**Figure S1**. The EDS spectra and atomic ratio of corresponding elements in AF-MCTS composite.


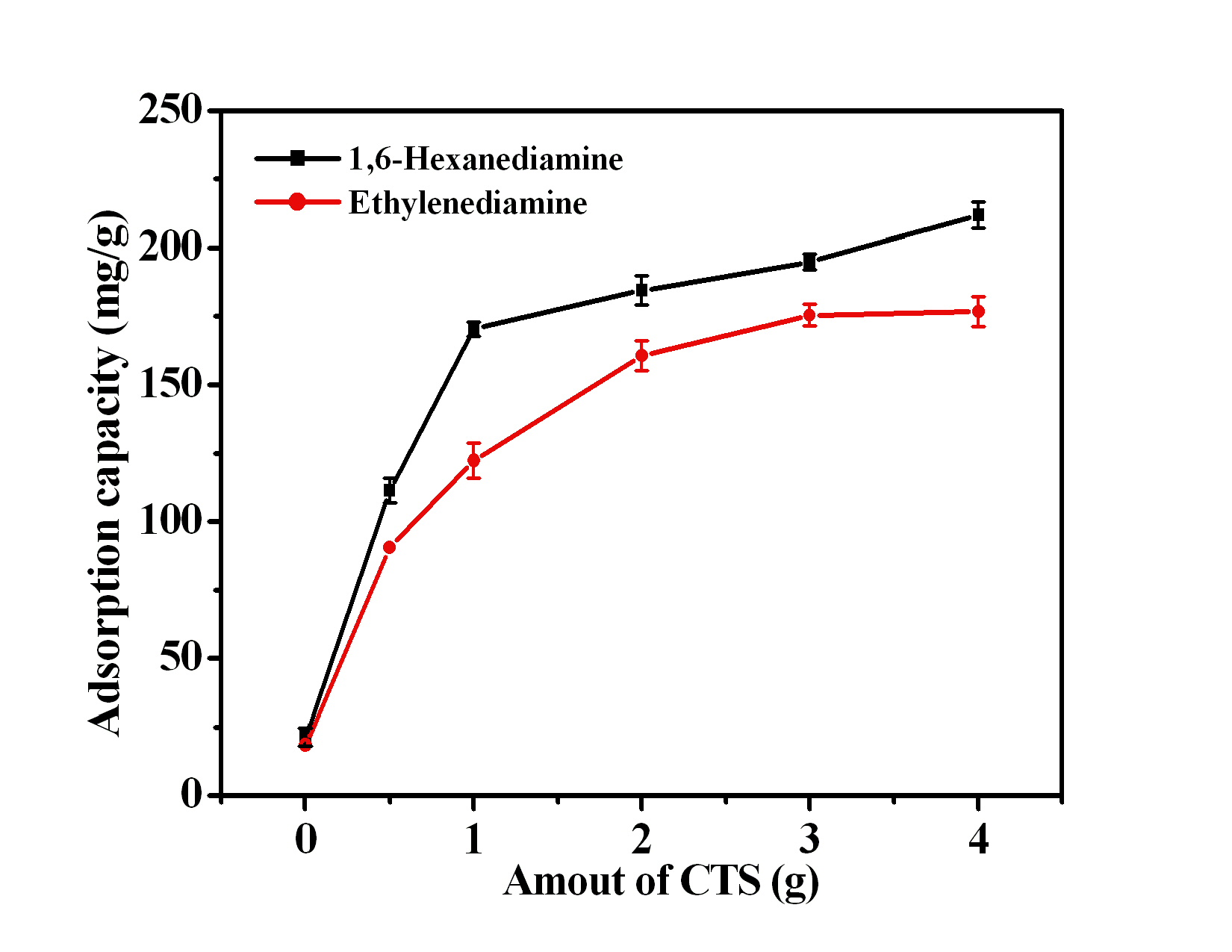


**Figure S2**. Effect of amount of CTS on adsorption of Cr(VI) by ethylenediamine modified magnetic chitosan microspheres and 1,6-hexanediamine modified magnetic chitosan microspheres. Experimental conditions: pH 3.0; 50 mL of 100 mg/L Cr(VI) solution; 5 mg adsorbent.


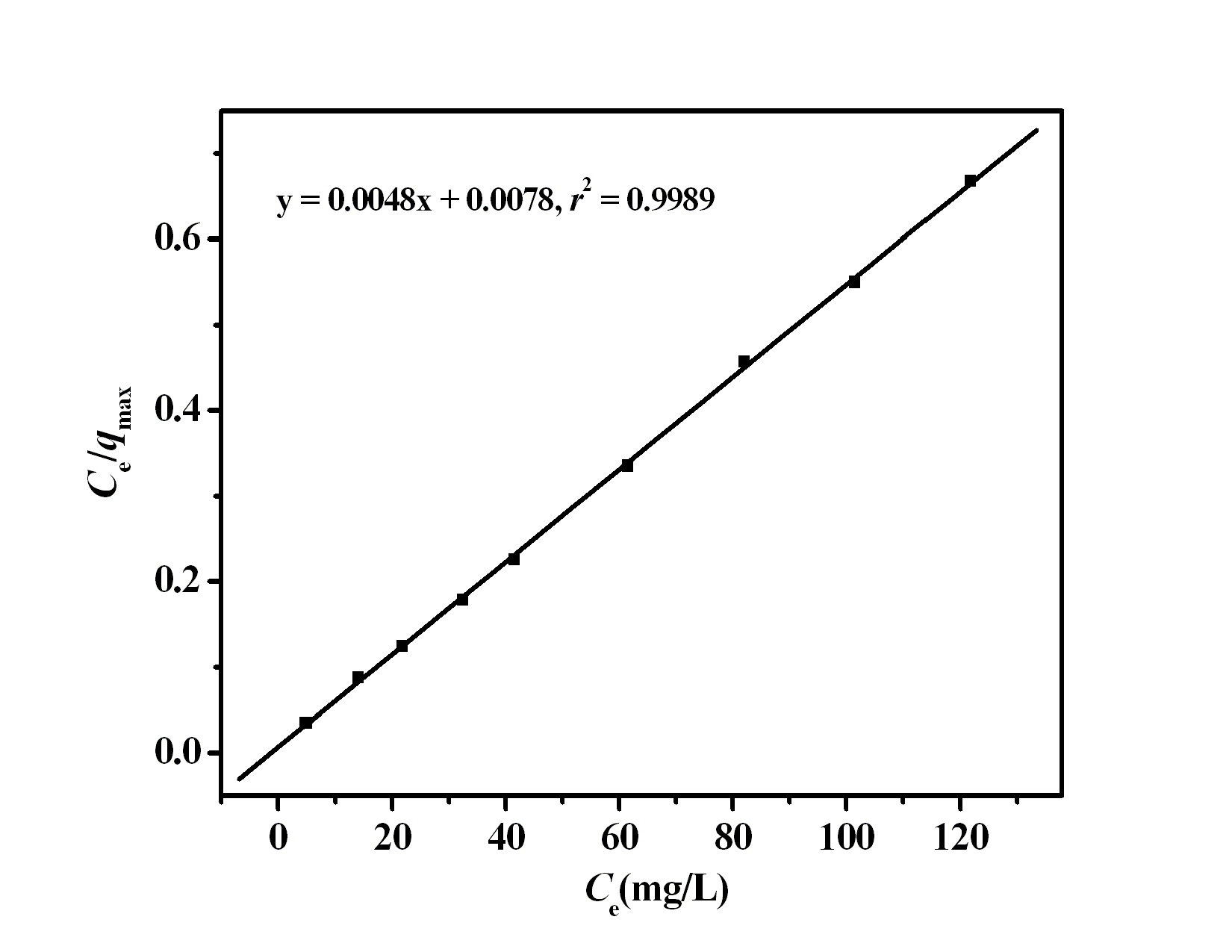

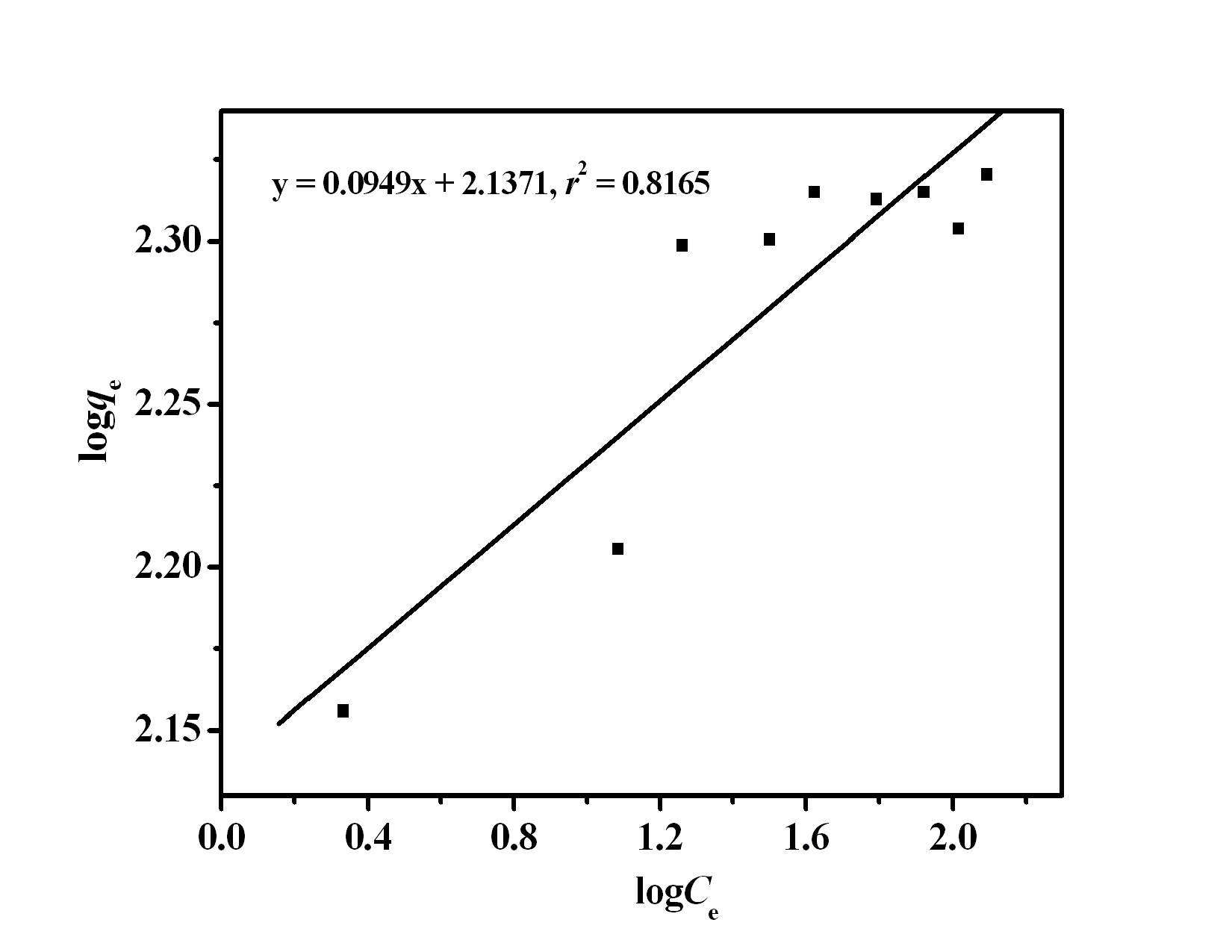


(A) (B)

**Figure S3.** Fitting curves of adsorption isotherm of Cr(VI) on AF-MCTS by Langmuir model (A) and Freundlich model (B).

**Figure S4**. Concentrations of Cr(VI) and Cr(III) in the residual solution after adsorption Cr(VI) on AF-MCTS. Reaction conditions: 5 mg of adsorbent; reaction temperature: 30 ^o^C; pH=3.0; initial Cr(VI) concentration: 100 mg/L.

**Figure S5**. Schematic diagram of regeneration and recyclability of the AF-MCTS for Cr(VI) removal.


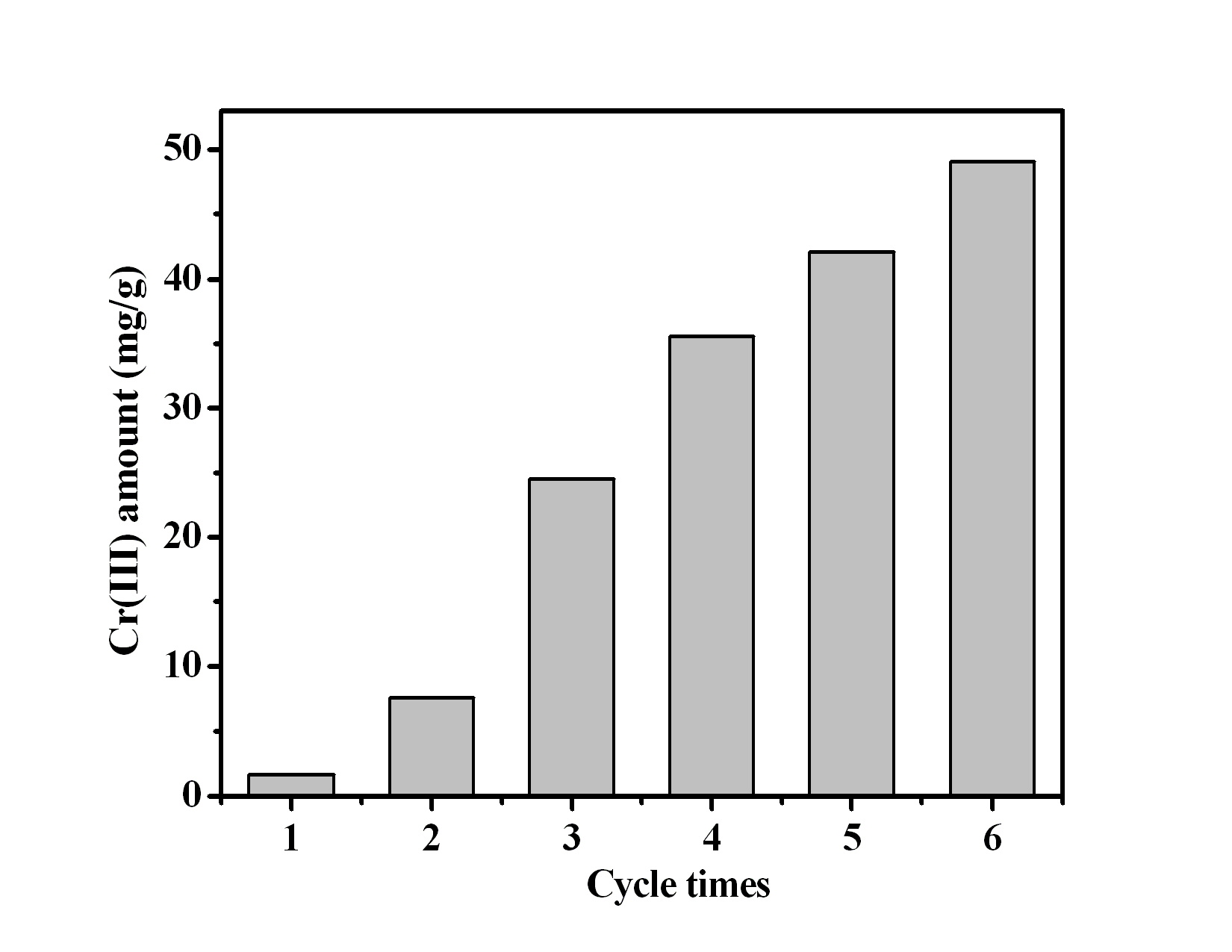


**Figure S6**. Cr(III) amount in the AF-MCTS after different cycle times.


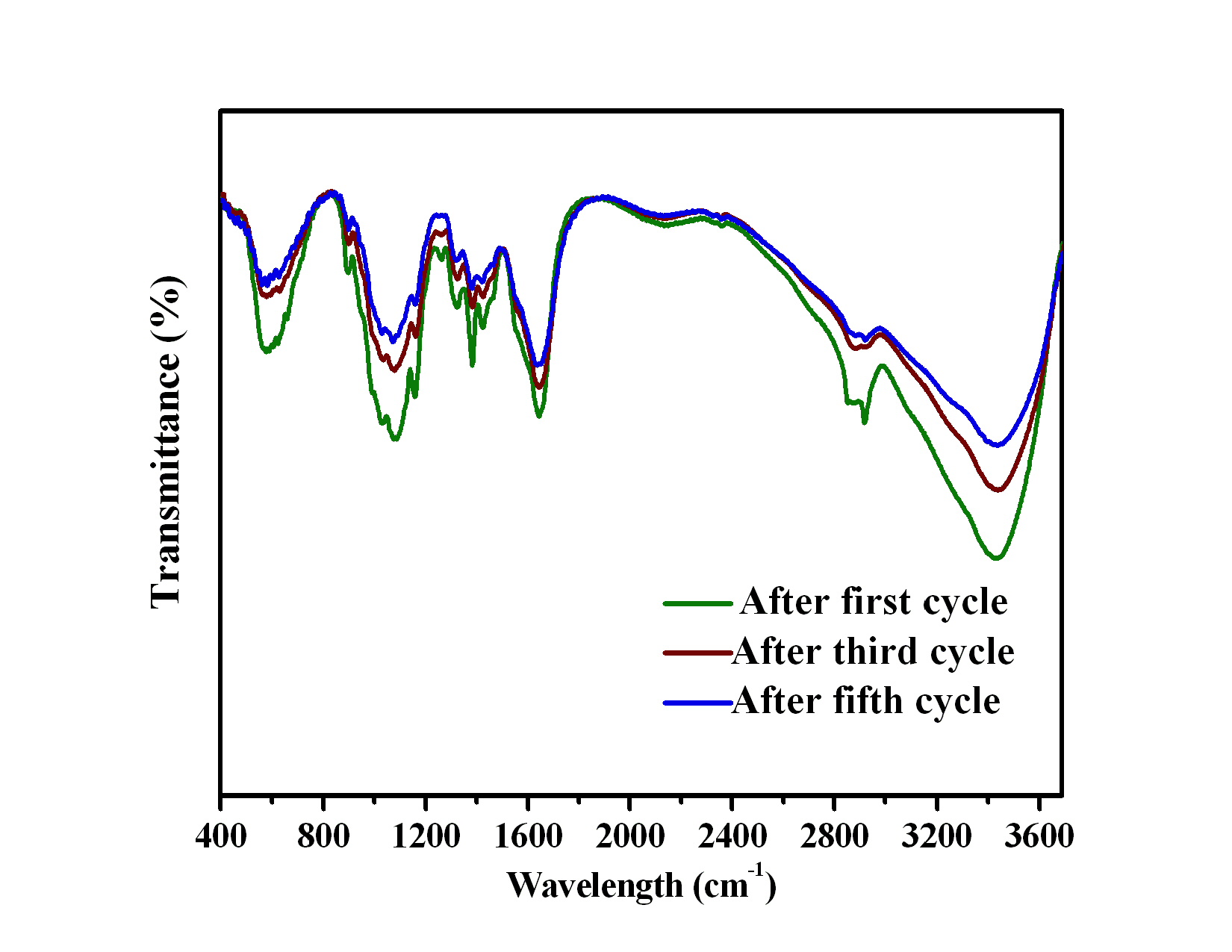


**Figure S7**. FT-IR spectra of the AF-MCTS after reuse.
